# Exposure to unpredictability and mental health: Validation of the brief version of the Questionnaire of Unpredictability in Childhood (QUIC-5) in English and Spanish

**DOI:** 10.3389/fpsyg.2022.971350

**Published:** 2022-11-09

**Authors:** Natasha G. Lindert, Megan Y. Maxwell, Sabrina R. Liu, Hal S. Stern, Tallie Z. Baram, Elysia Poggi Davis, Victoria B. Risbrough, Dewleen G. Baker, Caroline M. Nievergelt, Laura M. Glynn

**Affiliations:** ^1^Department of Psychology, Chapman University, Orange, CA, United States; ^2^Department of Statistics, University of California, Irvine, Irvine, CA, United States; ^3^Department of Anatomy/Neurobiology, University of California, Irvine, Irvine, CA, United States; ^4^Department of Pediatrics, University of California, Irvine, Irvine, CA, United States; ^5^Department of Neurology, University of California, Irvine, Irvine, CA, United States; ^6^Department of Psychology, University of Denver, Denver, CO, United States; ^7^Department of Psychiatry and Human Behavior, University of California, Irvine, Irvine, CA, United States; ^8^Center of Excellence for Stress and Mental Health, Veterans Affairs, San Diego, CA, United States; ^9^Department of Psychiatry, University of California, San Diego, San Diego, CA, United States

**Keywords:** early life adversity, unpredictability, mental health, anxiety, depression

## Abstract

Unpredictability is increasingly recognized as a primary dimension of early life adversity affecting lifespan mental health trajectories; screening for these experiences is therefore vital. The Questionnaire of Unpredictability in Childhood (QUIC) is a 38-item tool that measures unpredictability in childhood in social, emotional and physical domains. The available evidence indicates that exposure to unpredictable experiences measured with the QUIC predicts internalizing symptoms including depression and anxiety. The purpose of the present study was to validate English and Spanish brief versions (QUIC-5) suitable for administration in time-limited settings (e.g., clinical care settings, large-scale epidemiological studies). Five representative items were identified from the QUIC and their psychometric properties examined. The predictive validity of the QUIC-5 was then compared to the QUIC by examining mental health in four cohorts: (1) English-speaking adult women assessed at 6-months postpartum (*N* = 116), (2) English-speaking male veterans (*N* = 95), (3) English-speaking male and female adolescents (*N* = 155), and (4) Spanish-speaking male and female adults (*N* = 285). The QUIC-5 demonstrated substantial variance in distributions in each of the cohorts and is correlated on average 0.84 (*r’s* = 0.81–0.87) with the full 38-item version. Furthermore, the QUIC-5 predicted internalizing symptoms (anxiety and depression) in all cohorts with similar effect sizes (*r’s* = 0.16–0.39; all *p’s* < 0.05) to the full versions (*r’s* = 0.19–0.42; all *p’s* < 0.05). In sum, the QUIC-5 exhibits good psychometric properties and is a valid alternative to the full QUIC. These findings support the future use of the QUIC-5 in clinical and research settings as a concise way to measure unpredictability, identify risk of psychopathology, and intervene accordingly.

## Introduction

There is little dispute that early life experience imparts a lasting imprint on trajectories of health and development across the lifespan. A rapidly accumulating body of evidence has shown poverty, neglect and abuse as well as other adverse experiences in childhood influence morbidity and mortality, including increased risk for psychopathology (LeMoult et al., 2020; Luby et al., 2020; McLaughlin et al., 2020). Unfortunately, exposure to childhood adversity is common – it is estimated that in developed nations 40–60% of adults have experienced at least one adverse childhood experience and 15–25% have been exposed to 2 or more (Green et al., 2010; Kessler et al., 2010; Merrick et al., 2018; Hughes et al., 2021). Although global estimates are less well described, projections indicate that one billion children ages 2–17 (more than 50%) are the victims of physical, sexual or emotional abuse each year (Hillis et al., 2016). Not surprisingly, due to its widespread prevalence, the global impacts on human and fiscal capital are significant. For example, as many as 20–30% of mental health disorders are attributable to childhood adversity (Green et al., 2010; Kessler et al., 2010). Similarly, the total annual burden of adversity exposures in Europe and North America total over $1.3 billion, roughly equating to 3.5% of North America’s and 2.7% of Europe’s regional gross domestic products (Bellis et al., 2019).

Although childhood adversity has been widely studied, much of this work has focused on a canonical set of adverse childhood experiences or ACEs (Felitti et al., 1998; Hughes et al., 2017) and employs a cumulative risk approach in which these adverse experiences are summed to create a composite risk score without regard for type of exposure. This model, while playing a pivotal role in documenting the pervasive and persisting influences of adversity exposures, is limited in that although it successfully identifies risk at the population level, it is unable to identify risk at the level of the individual (Baldwin et al., 2021). This observation, coupled with a need to advance conceptual models focused on the developmental consequences of early life adversity, has spurred calls for a more nuanced approach in which childhood adversity is characterized in terms of multiple dimensions or underlying factors (Ellis et al., 2009; McLaughlin and Sheridan, 2016). These dimensional models do converge on core components including threat/harshness and deprivation (Ellis et al., 2009; Sheridan and McLaughlin, 2014; Berman et al., 2022). Increasing consensus also includes a focus on unpredictability as a distinct dimension worthy of investigation (Ellis et al., 2009; Baram et al., 2012; Glynn and Baram, 2019; McLaughlin et al., 2021; Liu and Fisher, 2022).

There are studies that have characterized unpredictability in a number of ways including parental behaviors (Ainsworth and Bowlby, 1991; Bernard et al., 2013; Davis et al., 2017, 2019), parental mood lability (Glynn et al., 2018; Howland et al., 2021), residential transitions, changes in parental cohabitation and employment status (Doom et al., 2016) and there are instruments that quantify specific components of unpredictability such as family routines and household chaos (Jensen et al., 1983; Matheny et al., 1995). However, until recently there did not exist a tool to assess the broad dimension of childhood unpredictability. To meet this need, we developed and validated the Questionnaire of Unpredictability in Childhood, or QUIC (Glynn et al., 2019), a 38-item self-report instrument which comprehensively assesses exposures to unpredictability in social, emotional and physical environments. The psychometric properties were originally examined in four independent cohorts and include excellent reliability for both the English and Spanish versions (Glynn et al., 2019; Liu et al., under review). Importantly, retrospective reports on the QUIC were linked to prospectively gathered measures of unpredictability in childhood validating the retrospective recall on this instrument. For example, 83% of adolescents who endorsed the item “I moved frequently” had in fact moved 3 or more times during their lifetimes. It also is the case that self-reports of more unpredictability assessed with the QUIC were associated with two prospectively measured observational measures of parental inputs – unpredictable sensory signals in infancy (Davis et al., 2017), and unpredictable maternal mood in infancy and childhood (Glynn et al., 2018) – providing support for the construct validity of the instrument.

Growing evidence also demonstrates the predictive validity of the QUIC as a tool to measure the associations between unpredictability and mental health risk. In our initial validation paper, the QUIC predicted anxiety and depression in three independent cohorts: adult females, male veterans and male and female adolescents (Glynn et al., 2019). Importantly, these associations persisted after accounting for other indicators of early life adversity including both threat and deprivation. The subsequent QUIC Spanish language validation study then also associated unpredictability assessed via the QUIC-SP to anxiety and depressive symptoms in adulthood (as well as anhedonia and poorer physical health; Liu et al., under review). Building upon these findings, the QUIC has now also been linked to posttraumatic stress, anhedonic, depressive and anxiety symptoms as well as suicidal ideation in trauma-exposed male veterans (Spadoni et al., 2022). Further, Gillespie and Rao (2022) recently documented associations between increased childhood unpredictability assessed with the QUIC with higher rates of depressive symptoms and compromised executive function in adolescents. Thus, increasing evidence suggests that childhood unpredictability is a distinct dimension of early life adversity that deserves additional consideration and that the QUIC is a valid tool to further these investigations.

While the QUIC is emerging as a comprehensive and valid tool to measure unpredictability in early life, a measure of this length may not be feasible in time-limited research and clinical contexts. Therefore, this study aimed to validate a brief version of the QUIC and QUIC-SP for use in these settings (QUIC-5 and QUIC-SP-5). We did this by examining the psychometric properties of these measures in four independent cohorts.

## Methods

### Procedure

The Questionnaire of Unpredictability in Childhood, which is validated in English (QUIC; Glynn et al., 2019) and Spanish (QUIC-SP; Liu et al., under review), is a 38-item questionnaire that measures unpredictability in childhood. The QUIC broadly assesses social, emotional, and physical environments and answers are given as yes/no responses. To create brief versions of this measure, five items were identified from the QUIC to form the QUIC-5 and QUIC-SP-5 (Table 1). These five items were selected by expert consensus to include experiences that represent different domains of unpredictability and that are applicable to children from birth to 18 years of age. Scores on the full QUICs range from 0 to 38, while scores on the QUIC-5 and QUIC-SP-5 range from 0 to 5. For all versions, a higher score indicates greater exposure to unpredictability in childhood. In this study, the QUIC-5 and QUIC-SP-5 scores were compared to the QUIC and QUIC-SP scores, respectively, to characterize the performance of the brief versions. Additionally, the correlation between mental health outcomes and the full QUICs and short QUICs were compared to assess the predictive validity of the brief version.

**TABLE 1:** Questionnaire of Unpredictability in Childhood-5 items.

English	Spanish
•Before age 12, I had a bedtime routine (e.g., my parents tucked me in, my parents read me a book, I took a bath).*	• Antes de los 12 años, tenía una rutina antes de acostarme a dormir (por ejemplo, mis padres me cobijaban, me leían un libro, yo tomaba un baño).*
• At least one of my parents was unpredictable.	• Al menos uno de mis padres era impredecible.
• One of my parents could go from calm to furious in an instant.	• Uno de mis padres podría pasar en un instante de la calma a la furia.
• My parents had a stable relationship with each other.*	• Mis padres tenían una relación estable entre ellos.*
• In my house things I needed were often misplaced so that I could not find them.	• En mi casa las cosas que necesitaba muchas veces no estaban en su lugar, y no las podía encontrar.

*Indicates a reverse-scored item.

### Participants

The utility and predictive validity of the QUIC-5 was assessed in four independent cohorts: (1) adult females, (2) male military veterans, (3) adolescents, and (4) Spanish-speaking adults. See Table 2 for demographics for each cohort.

**TABLE 2:** Description of study cohorts.

	Adult females (*N* = 116)	Male veterans (*N* = 95)	Adolescents (*N* = 226)	Spanish-speaking adults (*N* = 285)
Age [mean years (SD)]	30 (5.8)	35 (10.9)	14 (2.6)	–
0–18 years (%)	0.0	0.0	94.2	0.0
18–24 years (%)	29.3	5.3	5.8	19.6
25–34 years (%)	53.4	61.1	0.0	38.9
35–44 years (%)	17.2	16.8	0.0	25.6
45–54 years (%)	0.0	7.4	0.0	9.5
55–64 years (%)	0.0	7.4	0.0	5.3
65 + years (%)	0.0	2.1	0.0	1.1
Sex/Gender (% female)	100.0	0.0	52.0	58.0
**Race/Ethnicity (%)**				
White, non-Hispanic	35.3	63.0	41.5	27.0
Hispanic/Latino(a)	42.2	14.1	36.6	67.0
Asian	12.1	5.4	5.8	3.5
Black	3.4	10.9	3.6	2.1
Multi-ethnic	6.9	6.5	12.5	0.4
**Highest level of education (mean years)**				
Less than 6 years (%)	0.0	0.0		7.7
6–10 years (%)	2.6	0.0		6.7
11–15 years (%)	55.2	77.9		26.0
16–20 years (%)	38.8	22.1		53.3
21–25 years (%)	2.6	0.0		5.6
More than 25 years (%)	0.9	0.0		0.7
**Annual household income (mean USD)**				
Under $5,000 (%)	0.9		0.0	0.0
$5,000–$34,999 (%)	31.5		6.4	26.7
$35,000–$64,999 (%)	27.8		12.3	36.8
$65,000–$99,999 (%)	10.2		22.3	20.4
$100,000–$249,999 (%)	25.9		47.3	14.7
$250,000–$449,999 (%)	2.9		8.6	1.4
$450,000+(%)	0.9		3.2	0.0

Income data were not available for male veterans. Exact age was not available for Spanish-speaking adults.

#### Cohort 1: Adult females assessed postpartum

Cohort 1 consisted of 116 adult females (age: 18–44 years) participating in an ongoing longitudinal study on maternal and child health. These participants completed the QUIC and the 10-item Edinburgh Postnatal Depression Scale (EPDS), which is a widely used and validated measure of depressive symptoms (Cox et al., 1987). The EPDS was administered at 6 months postpartum.

#### Cohort 2: Male military veterans

Cohort 2 consisted of 95 male veterans (age: 24–70 years) participating in either a follow-up assessment from a prospective, longitudinal study of deployment trauma or the Center of Excellence for Stress and Mental Health TBI/PTSD biorepository data archives. Participants completed the QUIC and Patient Health Questionnaire-9 (PHQ-9), which is a well validated 9-item questionnaire assessing severity of depressive symptoms (Löwe et al., 2004).

#### Cohort 3: Adolescents

Cohort 3 comprised 226 adolescents (52% female; age: 10–23 years) participating in a longitudinal study of maternal and child health. Adolescent participants completed the QUIC, the 12-item Children’s Depression Inventory (CDI; Kovacs, 2011), and the 20-item State-Trait Anxiety Inventory for Children (STAIC; Spielberger et al., 1973).

#### Cohort 4: Spanish-speaking adults

Cohort 4 consisted of 285 Spanish-speaking adults (58% female) recruited for an online validation study of the QUIC-SP. In addition to the QUIC-SP, these participants completed the Beck Depression Inventory (BDI; Beck et al., 1996; Sanz et al., 2003), which is a widely-used 21-item questionnaire quantifying depressive symptoms in adults. Participants also reported on their anxiety symptoms using the 10-item State-Trait Anxiety Inventory (STAI; Spielberger et al., 1982, 1983).

### Data analyses

The distribution of QUIC-5 scores were first compared to examine variability in scores among the four cohorts. Next, Pearson correlations were used to determine the strength of the associations between the full and brief versions within each cohort. Last, the predictive validity of the QUIC-5 was compared to that of the full QUIC by examining bivariate correlations between the two versions of the QUIC and measures of anxiety and depressive symptoms.

## Results

### Distribution of scores

**FIGURE 1:**
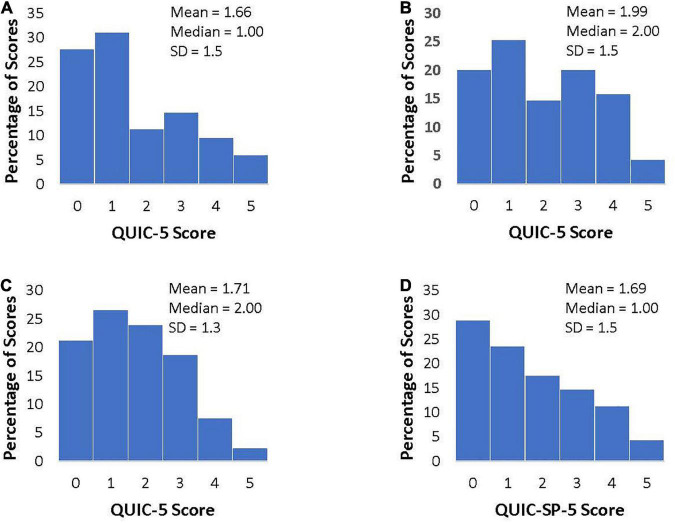
Distributions of QUIC-5 and QUIC-SP-5 Scores. **(A)** Adult females (*N* = 116), **(B)** male veterans (*N* = 95), **(C)** adolescents (*N* = 226), and **(D)** Spanish-speaking adults (*N* = 285).

The distributions, means, medians, and standard deviations of QUIC-5 scores for each cohort are shown in Figure 1. Compared to the adult females, adolescents, and Spanish-speaking adults, the male veterans had the highest QUIC-5 scores, which was consistent with the pattern of results observed with the full-length QUIC (Glynn et al., 2019).

### Endorsement rates and associations between brief and full versions

For each cohort, QUIC-5 scores were strongly correlated with full-length QUIC scores (all *r*’s > 0.81; all *p’s* < 0.001). Among the adult females, the QUIC-5 was correlated 0.82 (95% CI [0.75, 0.87]) with the QUIC, 0.85 (95% CI [0.78, 0.90]) among male veterans, and 0.81 (95% CI [0.76, 0.85]) among adolescents. Among Cohort 4 (Spanish-speaking adults), the QUIC-SP-5 and QUIC-SP were correlated 0.87 (95% CI [0.84, 0.90]).

### Predictive validity

**FIGURE 2:**
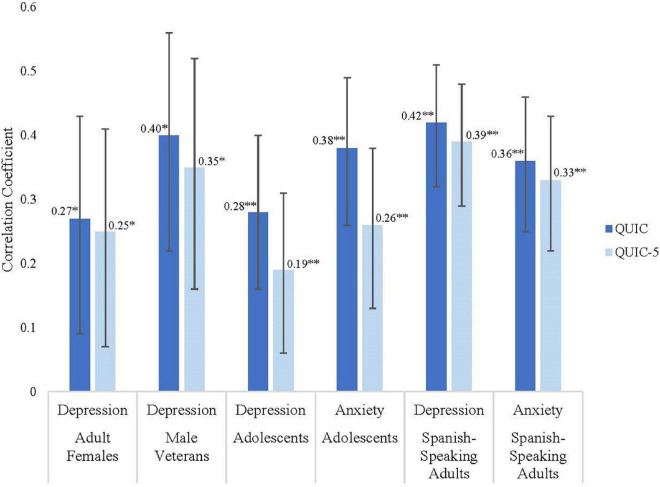
Predictive validity for mental health outcomes of the QUIC-5 compared to the QUIC (*p < 0.05; ^**^p < 0.01). Dark bars indicate the size of the correlation coefficient for the 38-item QUIC, and the light bars are the correlation for the QUIC-5. Bars indicate 95% confidence intervals.

The QUIC-5 and QUIC-SP-5 predicted internalizing symptoms (anxiety and depression) in each of the four cohorts (*r’s* = 0.16–0.39; all *p’s* < 0.05). Critically, the effect sizes were not substantively different from the association between the full-length QUIC and these same mental health indicators (*r’s* = 0.19–0.42; all *p’s* < 0.05; see Figure 2).

## Discussion

Here we show that both the English and Spanish language versions of the QUIC-5 demonstrate excellent promise as brief assessments of unpredictability in childhood. These 5-item versions are associated on average 0.84 with the full-length version across four independent cohorts that span sex/gender, socioeconomic, and cultural groups and range in age from adolescence through 70 years. This high correlation is especially promising given that the full version was validated against prospective assessments of early life unpredictability and exhibits strong content and discriminant validity (Glynn et al., 2019). Perhaps an even stronger indicator of the brief version’s promise is that among four diverse cohorts, the QUIC-5 predicts mental health outcomes with virtually identical effect sizes to the full-length QUIC.

Progress has been made in documenting the role of adversity in shaping health and development. However, a significant barrier that remains is the relative lack of neurobiological models testing biologically plausible mechanistic processes linking early life adversity to developmental and mental health outcomes (Smith and Pollak, 2021) – a gap that further investigations of unpredictability may help address (Birnie and Baram, 2022). Foundational studies on the formation of sensory circuits, such as vision and hearing established that modality-specific sequences of sensory signals at a moment-to-moment timescale are required for the maturation of these neural circuits and further that disrupted sequences of sensory signals (e.g., visual or auditory) during sensitive developmental periods disturb the sculpting and maturation of visual, somatosensory and auditory brain circuits, leading to commensurate functional deficits (Wiesel and Hubel, 1963; Khazipov et al., 2004; Hackett et al., 2011; Espinosa and Stryker, 2012). The mechanisms involve altered synaptic pruning by microglia (Bolton et al., 2022) as well as maturation and maintenance in brain circuits subserving cognitive and emotional functions (Avishai-Eliner et al., 2001; Brunson et al., 2005; Ivy et al., 2010; Singh-Taylor et al., 2015; Molet et al., 2016a,b; Bolton et al., 2022). These preclinical studies provide mechanistic insight into the processes by which sequences of signals shape the organization of the developing brain and are complemented by research in human infants characterizing the importance of moment-to-moment signals (Yu and Smith, 2013). Building upon these principles and working with human infants, a translational model was developed to test the hypothesis that sequences of parental signals on a moment-to-moment timescale influence the maturation of human brain circuits underlying cognitive and emotion functions (Davis et al., 2017). These investigations do indicate that exposure to unpredictable parental signals is associated with poorer memory, compromised self-regulation and altered brain development of circuits related to cognitive and emotion function in infants and children (Davis et al., 2017, 2019; Granger et al., 2021).

Given that unpredictability is emerging as a core dimension in multiple conceptual models of childhood adversity (Ellis et al., 2009; Baram et al., 2012; Glynn and Baram, 2019; McLaughlin et al., 2021), the brevity of and predictive validity of the QUIC-5 has the potential to further facilitate the investigation of this dimension in a wider range of research studies. In addition to its increased utility in research settings, the QUIC-5 has potential as a brief and feasible tool for implementation in clinical contexts. A growing number of leading health care organizations now advocate for universal screening for childhood adversity in primary care resulting in increased implementation (AAP, 2017; Dube, 2018; Centers for Disease Control and Prevention, 2019; Hetherington, 2020). However, there are barriers to this implementation on the part of the clinical care providers, including time constraints, discomfort related to querying about sensitive items, uncertainty regarding appropriate referrals and potential iatrogenic effects (Maunder et al., 2020; Thompson et al., 2020). One potential advantage of screening for unpredictability is that the questions are relatively innocuous and many of these aspects of the family environment are amenable to change without requiring significant resources.

Research thus far indicates that the effect sizes for the associations between unpredictability with mental health outcomes are similar to those of previously established adverse childhood experiences such as poverty and abuse (Lawson et al., 2018; LeMoult et al., 2020), underscoring the neccessity of continuing to work on this crucial aspect of the early environment. It has been estimated that a 10% reduction in childhood adversity exposures in Europe and North America could equate to a savings of $105 billion or 3 million disability-adjusted life-years annually (Bellis et al., 2019). Multiple aspects of unpredictability (e.g., bedtime or mealtime routines, consistency in parenting practices) are sensitive to intervention and do not require significant monetary resources nor change to societal structural inequalities for remedy, unlike many other sources of childhood adversity. This is not to suggest that efforts should not be made to address the glaring disparities in adversity exposures arising from structural inequalities or other more intractable adversities, but rather that addressing unpredictability is an additional avenue to support the health of children. To this end, the QUIC and QUIC-5 can aid in endeavors to mitigate the deleterious influences of exposure to adversity in early life, with the potential to reduce the associated burdens on both monetary and human capital and improve public health.

## Data availability statement

The data presented in the study are available upon request to the corresponding author.

## Ethics statement

The studies involving human participants were reviewed and approved by University of California, Irvine IRB; San Diego, VA IRB. Written informed consent to participate in this study was provided by all adult study participants. Written informed consent for minors to participate in this study was provided by the participants’ legal guardian/next of kin. Additionally, children provided assent to participate in this study.

## Author contributions

LG, TB, and HS developed the QUIC-5. LG and NL conducted the statistical analyses. LG, NL, MM, and SL drafted the manuscript. LG, EP, TB, HS, VR, DB, and CN participated in study conception and design and contributed funding. All authors provided editorial feedback and approved the submitted version.
