# Addendum: Exposure to unpredictability and mental health: Validation of the brief version of the Questionnaire of Unpredictability in Childhood (QUIC-5) in English and Spanish

**DOI:** 10.3389/fpsyg.2023.1241626

**Published:** 2023-07-25

**Authors:** Natasha G. Lindert, Megan Y. Maxwell, Sabrina R. Liu, Hal S. Stern, Tallie Z. Baram, Elysia Poggi Davis, Victoria B. Risbrough, Dewleen G. Baker, Caroline M. Nievergelt, Laura M. Glynn

**Affiliations:** ^1^Department of Psychology, Chapman University, Orange, CA, United States; ^2^Department of Statistics, University of California, Irvine, Irvine, CA, United States; ^3^Department of Anatomy/Neurobiology, University of California, Irvine, Irvine, CA, United States; ^4^Department of Pediatrics, University of California, Irvine, Irvine, CA, United States; ^5^Department of Neurology, University of California, Irvine, Irvine, CA, United States; ^6^Department of Psychology, University of Denver, Denver, CO, United States; ^7^Department of Psychiatry and Human Behavior, University of California, Irvine, Irvine, CA, United States; ^8^Center of Excellence for Stress and Mental Health, Veterans Affairs, San Diego, CA, United States; ^9^Department of Psychiatry, University of California, San Diego, San Diego, CA, United States

**Keywords:** early life adversity, unpredictability, mental health, anxiety, depression

Due to requests for the items, instructions, and scoring information used in this study, the authors have now provided this material as Supplementary Datasheet 1 which is now published alongside the original article.

In the published article, there were also errors in **Table 1**. Item 1, in English, stated “Before age 11, I had a bedtime routine (e.g., my parents tucked me in, my parents read me a book, I took a bath).” The correct statement is “Before age 12, I had a bedtime routine (e.g., my parents tucked me in, my parents read me a book, I took a bath).” Item 1, in Spanish, stated “Antes de los 11 años, tenía una rutina antes de acostarme a dormir (por ejemplo, mis padres me cobijaban, me leían un libro, yo tomaba un baño).” The correct statement is “Antes de los 12 años, tenía una rutina antes de acostarme a dormir (por ejemplo, mis padres me cobijaban, me leían un libro, yo tomaba un baño).” Item 3, in Spanish, stated “Uno de mis padres podría pasar en un instante de la calma al estrés y los nervios.” The correct statement is “Uno de mis padres podría pasar en un instante de la calma a la furia.” Item 4, in Spanish, stated “Mis padres tenían una relación estable.” The correct statement is “Mis padres tenían una relación estable entre ellos.” The corrected Table 1 and its caption appear below.

**Table 1:** Questionnaire of Unpredictability in Childhood-5 items.

**English**	**Spanish**
Before age 12, I had a bedtime routine (e.g., my parents tucked me in, my parents read me a book, I took a bath).^*^	Antes de los 12 años, tenía una rutina antes de acostarme a dormir (por ejemplo, mis padres me cobijaban, me leían un libro, yo tomaba un baño).^*^
At least one of my parents was unpredictable.	Al menos uno de mis padres era impredecible.
One of my parents could go from calm to furious in an instant.	Uno de mis padres podría pasar en un instante de la calma a la furia.
My parents had a stable relationship with each other.^*^	Mis padres tenían una relación estable entre ellos.^*^
In my house things I needed were often misplaced so that I could not find them.	En mi casa las cosas que necesitaba muchas veces no estaban en su lugar, y no las podía encontrar.

^*^Indicates a reverse-scored item.

The authors apologize for these errors and state that this does not change the scientific conclusions of the article in any way. The original article has been updated.

