# Supplementary material for: Exposure to unpredictability and mental health: Validation of the brief version of the Questionnaire of Unpredictability in Childhood (QUIC-5) in English and Spanish
Source: Front Psychol. 2022 Nov 9;13:971350. doi: 10.3389/fpsyg.2022.971350 (PMC9682115; doi:10.3389/fpsyg.2022.971350)
Supplement: Supplementary file 1 [file Data_Sheet_1.docx]

# Appendix A

# Self-Report (12+)

**Questionnaire of Unpredictability in Childhood – Brief (QUIC-5)**

**Instructions and Items:**

This set of questions asks about your childhood experiences. When we say parents, we mean whoever in your life fills that role for you (e.g., biological parents, step-parents, grandparents, foster parents). This could be one person, or this could be multiple people.

We are going to ask about your experiences from birth to age 18 (or your whole life, if you are less than 18 years old). These answers should be based on your own memories, not on things you later learned from your parents or others.

**Please answer these questions based on your typical or average experiences during childhood.**

|  | **Yes** | **No** |
| --- | --- | --- |
| 1. Before age 12, I had a bedtime routine (e.g., my parents tucked me in, my parents read me a book, I took a bath). | **1** | **0** |
| 1. At least one of my parents was unpredictable. | **1** | **0** |
| 1. One of my parents could go from calm to furious in an instant. | **1** | **0** |
| 1. My parents had a stable relationship with each other. | **1** | **0** |
| 1. In my house, things I needed were often misplaced so that I could not find them. | **1** | **0** |

**Scoring Information:**

The QUIC-5 Self-Report (12+) consists of a single score. A higher score indicates more exposure to unpredictability in childhood. To obtain the score, reverse score select items (indicated by an R after the item number) and then calculate the sum of the items:

QUIC-5 Score = 1R + 2 + 3 + 4R + 5

**Reference:**

Lindert, N.G., Maxwell, M.Y., Liu, S.R., Stern, H.S., Baram, T.Z., Davis, E.P., Risbrough, V.B., Baker, D.G., Nievergelt, C.M., & Glynn, L.M. Exposure to unpredictability and mental health: Validation of the brief version of the Questionnaire of Unpredictability in Childhood (QUIC-5) in English and Spanish. *Frontiers in Psychology.* 13:971350 (2022). doi: 10.3389/fpsyg.2022.971350.

# Appendix B

**Self-Report (12+) – Spanish**

**Questionnaire of Unpredictability in Childhood – Brief (QUIC-SP-5)**

**Instrucciones y Artículos:**

Este es un conjunto de preguntas sobre sus experiencias en la niñez. Cuando decimos padres, nos referimos a cualquier persona que desempeñe ese papel en su vida (por ejemplo, padres biológicos, padrastros, abuelos, padres de crianza temporal). Esto puede ser una persona, o estos pueden ser varias personas.

Le vamos a preguntar sobre sus experiencias desde su nacimiento a la edad de 18 (o toda su vida, si usted es menor de 18 años de edad). Estas respuestas deben ser basadas en sus propios recuerdos, no en cosas que usted aprendió más tarde de sus padres u otros.

**Por favor responda a estas preguntas basándose en sus experiencias típicas o promedio durante su niñez.**

|  | **Sí** | **No** |
| --- | --- | --- |
| 1. Antes de los 12 años, tenía una rutina antes de acostarme a dormir (por ejemplo, mis padres me cobijaban, me leían un libro, yo tomaba un baño). | **1** | **0** |
| 1. Al menos uno de mis padres era impredecible. | **1** | **0** |
| 1. Uno de mis padres podría pasar en un instante de la calma a la furia. | **1** | **0** |
| 1. Mis padres tenían una relación estable entre ellos. | **1** | **0** |
| 1. En mi casa, las cosas que necesitaba muchas veces no estaban en su lugar, y no las podía encontrar. | **1** | **0** |

**Scoring Information:**

The QUIC-SP-5 Self-Report (12+) consists of a single score. A higher score indicates more exposure to unpredictability in childhood. To obtain the score, reverse score select items (indicated by an R after the item number) and then calculate the sum of the items:

QUIC-SP-5 Score = 1R + 2 + 3 + 4R + 5

**Información de Puntaje:**

El QUIC-SP-5 Self-Report (12+) consiste en un puntaje. Un puntaje más alto indica más exposición a la imprevisibilidad en la niñez. Para obtener el puntaje, seleccione los artículos de puntaje inverso (indicados por una R después del número del artículo) y después calcule la suma de los artículos:

QUIC-SP-5 Puntaje = 1R + 2 + 3 + 4R + 5

**Reference/Referencia:**

Lindert, N.G., Maxwell, M.Y., Liu, S.R., Stern, H.S., Baram, T.Z., Davis, E.P., Risbrough, V.B., Baker, D.G., Nievergelt, C.M., & Glynn, L.M. Exposure to unpredictability and mental health: Validation of the brief version of the Questionnaire of Unpredictability in Childhood (QUIC-5) in English and Spanish. *Frontiers in Psychology*.13:971350 (2022). doi: 10.3389/fpsyg.2022.971350.
